# The Diagnosis of Epidemic Meningitis and the Control of Its Treatment by Rapid Bacteriologic and Serologic Methods

**Published:** 1919

**Authors:** 


### *The Diagnosis of Epidemic Meningitis and the Control of its Treatment by Rapid Bacteriologic and Serologic Methods.*

By Lieutenants M. B. Cohen and J. S. Fleming. *Journal of Infectious Diseases,* October 1918.

Meningitis is an acute disease which requires careful diagnosis and treatment, and it is imperative that the laboratory, thoroughly equipped and in skilled hands, be called on for aid at the earliest possible moment.

The meningococcus is not an aerobic bacillus but a partial tension organism, requiring for perfect growth a percentage of oxygen only slightly less than air contains. Bearing this fact in mind meningococcus culture becomes extremely simple. It can be isolated on any enriched medium, such as blood agar. The reaction may be as high as plus 1.0 acid to phenolthalein.

Partial tension cultures are made by attaching a slant of meningococcus material to a fresh agar slant of B. subtilis, the latter using up sufficient oxygen to make the gaseous medium of the proper oxygen tension for meningococcus growth. This has been clearly proven by experiments.

Herrick states that epidemic meningitis begins as a meningococcus sepsis, and, while there is no definite localization, it is imperative to make a diagnosis in order to prevent the onset of meningitis. Partial tension cultures should be made from the clear spinal fluid. It is of the utmost importance also to make blood cultures. Baeslack and associates found meningococci in the blood of 36 o/o of early cases.

In well-developed cases the organism can be demonstrated in direct smears from spinal fluid. The centrifuged fluid is planted on 4 or 5 tubes of human blood agar, then placed in a jar with $Na_2CO_3$ in the bottom and 10 o/o sulphuric acid. After twenty-four hours the cultures will be found to have grown profusely. Cultures of 15 spinal fluids showing Gram-negative diplococci, cultures in $CO_2$, gave positive cultures in 13 of the cases.

*Summary.*

The meningococcus is a microaerophil.

The optimum reduction for its culture has been measured.

Simple, rapid and accurate methods of isolation and cultivation are described.

It grows best in an atmosphere of 10 o/o $CO_2$ and 90 o/o air.

In 500 nasopharyngeal cultures, 10.8 o/o carriers were found by $CO_2$, and 1.4 o/o by usual methods.

